# AI-Driven Monitoring for Fish Welfare in Aquaponics: A Predictive Approach

**DOI:** 10.3390/s25196107

**Published:** 2025-10-03

**Authors:** Jorge Saúl Fandiño Pelayo, Luis Sebastián Mendoza Castellanos, Rocío Cazes Ortega, Luis G. Hernández-Rojas

**Affiliations:** 1 Facultad de Ciencias Naturales e Ingeniería, Unidades Tecnológicas de Santander (UTS), Bucaramanga 680005, Colombia; jfandino@correo.uts.edu.co (J.S.F.P.); rcazes@correo.uts.edu.co (R.C.O.); 2 Facultad de Ingeniería, Universidad Autónoma de Bucaramanga (UNAB), Bucaramanga 680005, Colombia; lmendoza630@unab.edu.co; 3 Tecnológico de Monterrey, School of Engineering and Sciences, Monterrey 64849, N.L., Mexico

**Keywords:** aquaponics, environmental sensors, machine learning, random forest, neural networks, water quality monitoring, fish welfare classification, smart sensing systems, embedded monitoring, predictive analytics

## Abstract

This study addresses the growing need for intelligent monitoring in aquaponic systems by developing a predictive system based on artificial intelligence and environmental sensing. The goal is to improve fish welfare through the early detection of adverse water conditions. The system integrates low-cost digital sensors to continuously measure key physicochemical variables—pH, dissolved oxygen, and temperature—using these as inputs for real-time classification of fish health status. Four supervised machine learning models were evaluated: linear discriminant analysis (LDA), support vector machines (SVMs), neural networks (NNs), and random forest (RF). A dataset of 1823 instances was collected over eight months from a red tilapia aquaponic setup. The random forest model yielded the highest classification accuracy (99%), followed by NN (98%) and SVM (97%). LDA achieved 82% accuracy. Performance was validated using 5-fold cross-validation and label permutation tests to confirm model robustness. These results demonstrate that sensor-based predictive models can reliably detect early signs of fish stress or mortality, supporting the implementation of intelligent environmental monitoring and automation strategies in sustainable aquaponic production.

## 1. Introduction

Coupled aquaponics is an integrated system that combines aquaculture and hydroponics in a single closed cycle. In this system, the waste generated by the fish provides nutrients to the plants, and the plants, in turn, filter the water that returns to the aquatic system. This model is more efficient than uncoupled aquaponics, in which the fish and plant culture systems are separated, leading to greater complexity in nutrient and water quality management [1,2,3]. In coupled aquaponics, because both processes are interdependent, the use of water and nutrients is optimized, creating a more balanced and sustainable cycle [1,2]. Furthermore, coupled aquaponic systems have the potential to enhance fish welfare by maintaining stable environmental conditions, reducing stress, and preventing disease outbreaks [4,5]. Ensuring fish welfare is crucial in aquaponic systems, as it directly affects growth rates, disease resistance, and overall productivity [6,7].

Maintaining optimal physicochemical parameters—such as pH, temperature, and dissolved oxygen—is critical in coupled aquaponic systems, as imbalances in these variables can negatively affect both nutrient availability and fish welfare. Studies have shown that fluctuations in pH can alter the solubility and bioavailability of nutrients, compromising plant uptake and increasing physiological stress in fish [8,9]. Likewise, inadequate dissolved oxygen levels are associated with reduced fish growth, immunosuppression, and higher mortality rates [10]. Temperature also plays a crucial role, affecting metabolic activity, microbial dynamics, and the efficiency of nitrification processes [11]. Therefore, controlling these key variables ensures not only nutrient stability but also the overall resilience of aquaponic systems [12]. Recent advances emphasize the importance of real-time monitoring and predictive analytics to maintain these parameters within optimal thresholds and prevent critical disruptions to fish health and system productivity [13,14].

Traditionally, the monitoring and control of these variables have been conducted using manual methods or simple automated systems. However, these approaches often lack the ability to predict and proactively respond to critical system conditions. The use of artificial intelligence (AI) models represents a promising solution to overcome these limitations, providing accurate predictions and early warnings of potential mismatches in aquatic conditions [15,16]. AI and machine learning (ML) techniques have been widely adopted in agriculture to optimize processes, manage resources efficiently, and enhance sustainability. Recent studies have demonstrated the potential of ML and deep learning (DL) in areas such as water and nutrient management, pest control, and climate adaptation [17]. Several studies have explored the application of AI in aquaponic systems. Machine learning techniques such as artificial neural networks (ANNs), fuzzy logic systems, and decision trees have been implemented to optimize nutrient management and improve water quality control [16,18]. For instance, recent research has demonstrated the effectiveness of AI-driven models in forecasting dissolved oxygen levels, optimizing pH balance, and enhancing temperature regulation in aquaponics [16]. Furthermore, AI-based control strategies have been integrated with Internet of Things (IoT) technology to enable real-time monitoring and adaptive decision-making, which contributes to both system efficiency and fish welfare [18].

Recent applications extend beyond environmental control to the direct monitoring of fish welfare. For example, machine vision and AI techniques are being developed to non-invasively assess indicators such as behavior, morphology, and biomass in aquaculture, enabling the real-time health assessment and early detection of stress or disease [19,20].

Despite these advancements, challenges remain in achieving highly reliable predictive models that can generalize across different aquaponic setups and environmental conditions. Most existing studies focus on optimizing physicochemical parameters or enhancing plant productivity, without directly linking these factors to biological indicators in fish. This gap limits the ability of aquaponic systems to proactively respond to critical events that compromise system productivity and sustainability.

This study proposes the development of a predictive model based on artificial intelligence for the monitoring and control of physicochemical conditions in coupled aquaponics. The objective is to prevent situations that may induce stress or mortality in fish, thereby safeguarding their welfare, using machine learning techniques such as support vector machines (SVMs), softmax neural networks, genetic algorithms, and random forest [15]. By incorporating insights from previous research, this approach seeks to optimize the efficiency of the aquaponic system, improve its sustainability, and increase productivity through a predictive alert system that responds to possible critical changes in water conditions.

## 2. Materials and Methods

### 2.1. Aquaponic System

The aquaponic system developed in this study consists of cress lettuce (*Lactuca sativa*) and red tilapia (*Oreochromis* spp.), operating in a closed circuit where fish waste is transformed into nutrients for the plants. In turn, the plants improve water quality before the water returns to the fish tank.

**Figure 1:**
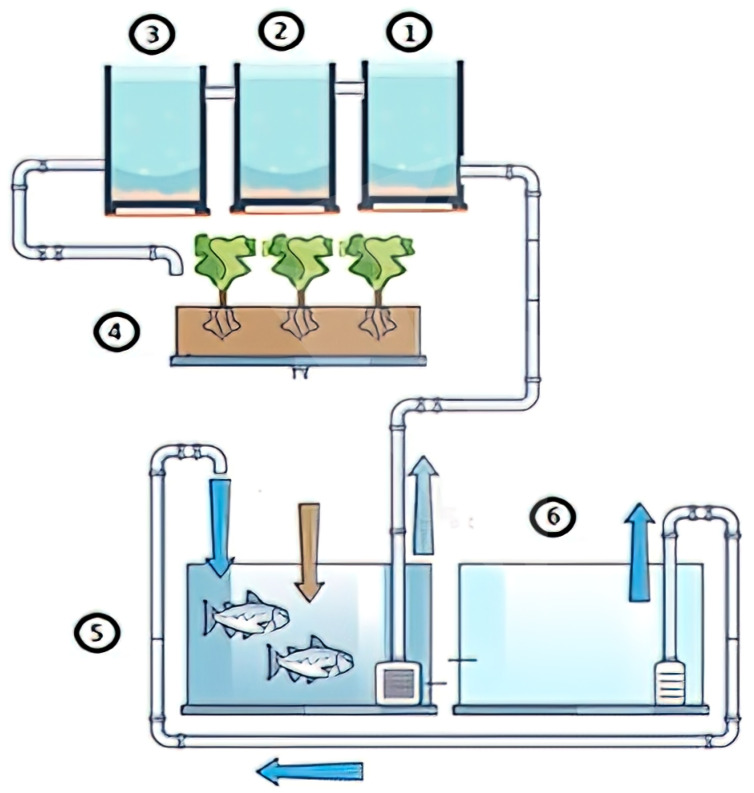
Diagram of the aquaponic system with its main components: (1) coarse sediments, (2) fine sediments, (3) biofilter, (4) plant bed, (5) fish tank, (6) cold water tank. The diagram was prepared by the authors.

Figure 1 illustrates the main components of the system, with each playing a crucial role in maintaining a balanced environment. The process begins in the fish tank (5), where red tilapia generate nutrient-rich water that is pumped into the filtration system. First, the coarse sediment tank (1) removes larger solid waste particles, preventing clogging and excessive accumulation. The water then flows into the fine sediment tank (2), which captures smaller residues not eliminated in the previous stage, thereby further refining the filtration process. Next, the biofilter (3) facilitates biological filtration, hosting microorganisms that convert fish waste—specifically ammonia—into nitrites and subsequently into nitrates, which serve as essential nutrients for plant growth. These nitrates are then absorbed by lettuce plants in the plant bed (4), effectively purifying the water before it recirculates back to the fish tank to complete the cycle.

To ensure optimal water quality for both fish and plants, three sensors were installed inside the fish tank (5). The pH sensor (model PH4502C; DFRobot, Shanghai, China) measures the acidity or alkalinity of the water within a range of 0 to 14, using a hydroponic sensing module with a BNC electrode probe. The dissolved oxygen sensor (model 183-ODPORT; Atlas Scientific, New York, NY, USA) provides accurate readings of oxygen concentration, which is critical for the respiration of aquatic organisms. Additionally, the water temperature was monitored with a digital sensor (model DS18B20; Maxim Integrated, San Jose, CA, USA), equipped with a waterproof probe and transducer, allowing precise monitoring of thermal variations within the tank.

According to the respective datasheets, the pH sensor PH4502C (DFRobot, Shanghai, China) has an accuracy of ±0.1–0.2 pH units and a resolution of 0.01 pH; the dissolved oxygen sensor 183-ODPORT (Atlas Scientific, New York, NY, USA) reports an accuracy of ±0.3 mg/L with a resolution of 0.1 mg/L; and the temperature sensor DS18B20 (Maxim Integrated, San Jose, CA, USA) offers ±0.5 °C accuracy in the range of –10 °C to +85 °C, with a resolution of 0.0625 °C.

#### 2.1.1. Temperature Control System

The temperature control system maintained water temperature within the appropriate range for red tilapia and lettuce, which is between 24 and 30 °C [21,22]. A DS18B20 water temperature sensor (Maxim Integrated, San Jose, CA, USA) monitored variations, triggering a pump motor (Shenzhen Jecod Co., Ltd., Shenzhen, China) connected to a cold-water tank when the temperature exceeded this range to cool the system [21]. Conversely, when the temperature dropped below the established threshold, an Eheim Thermocontrol E 300 heater (Eheim GmbH & Co. KG, Deizisau, Germany) was activated to restore suitable conditions and ensure thermal stability within the aquaponic system [23].

#### 2.1.2. Aerators and pH Regulators

Water oxygenation was achieved through the natural fall of water from the floating bed into the fish tank, along with three submersible aerators that activated when dissolved oxygen levels dropped below the appropriate range of 5–8 mg/L [24]. To regulate pH and prevent fluctuations in acidity, a peristaltic pump was used to dose organic components—specifically bicarbonate to increase pH and apple cider vinegar to lower it—maintaining pH within the 6.5–8.5 range and ensuring stable conditions within the system [25].

### 2.2. Experimental Protocol

This study was conducted using an aquaponic system designed for the cultivation of red tilapia (*Oreochromis* sp.), where three key environmental variables—pH, dissolved oxygen, and temperature—were continuously monitored, along with the systematic observation of fish condition over time.

The experiment lasted eight months, encompassing the full growth cycle of red tilapia, from fry (approximately 2 g) to adult stages (exceeding 400 g). This extended duration allowed the model to capture physiological and environmental variability associated with different life stages. During this period, water quality parameters were continuously recorded, as they are known to influence fish metabolism, immune function, and survival. The physiological condition of the fish was determined through regular observation of stress indicators, abnormal behaviors, and confirmed mortality events. These behavioral labels served as the ground truth for training the predictive models to recognize patterns associated with health deterioration throughout the production cycle.

Environmental variability was not experimentally induced. Although the system was equipped with automated aeration and circulation mechanisms, stress events emerged naturally during routine operation. These included episodes of oxygen depletion or temperature elevation, particularly following biomass increases, partial filter clogging, or feeding imbalances. While the aerators responded automatically to certain fluctuations, in several instances manual interventions—such as filter cleaning or biomass redistribution—were necessary to restore optimal conditions. Importantly, these corrective actions were implemented only after stress symptoms had already been observed, allowing the dataset to capture authentic physiological responses.

Fish health states (Healthy, Stress, Mortality) were labeled by two trained observers with experience in aquaponics. The observers were blinded to sensor readings and assigned labels based on direct visual assessment of behavioral and physiological cues (e.g., abnormal swimming, surface gasping, color changes) within a 30–60 min window preceding each sensor record. This approach ensured unbiased ground-truth definition under real aquaponic conditions and follows established practices for non-invasive welfare monitoring in fish [4,5,7].

The defined labeling protocol was consistently applied throughout the data collection phase, as detailed in Section 2.3.2.

Table 1 details the calibration routines and operational parameters of the monitoring setup. Weekly adjustment of the pH probe with buffer standards, verification of temperature against a glass thermometer, and cross-checking of dissolved oxygen with a YSI reference meter ensured stable and traceable measurements. The single-tank configuration (800 L) with a recirculation flow of ∼180 L/h provided consistent hydrodynamics, while the hourly sampling frequency defined the temporal resolution of the dataset. Together, these specifications constrain the level of measurement uncertainty and contextualize the conditions under which the predictive models were trained and validated.

### 2.3. Data Collection

#### 2.3.1. Key Variables Monitored

During the study, the following variables were recorded to monitor the health and efficiency of the aquaponic system. The pH of the water was measured as an indicator of its acidity or alkalinity, essential for maintaining chemical balance suitable for both fish and plants. Dissolved oxygen levels were monitored due to their fundamental role in fish respiration and the overall biological stability of the system. Temperature was tracked closely, given its critical influence on fish metabolism and plant growth rates. Additionally, the condition of the fish was evaluated through a direct visual assessment, considering behavioral patterns and physical appearance as indicators of their well-being.

#### 2.3.2. Sampling and Collection Time

The data collection process followed a structured methodology. The selection of measurement days was carried out randomly to ensure unbiased sampling across different system conditions, including both stable and variable periods. This randomness was essential to avoid systematic bias and train models capable of generalizing across diverse scenarios. The sampling frequency involved taking measurements at various times throughout the full 24-h cycle of each selected day, allowing for the analysis of both diurnal and nocturnal variations in key parameters. Over the course of the 8-month study, a total of 1823 data points were recorded, with measurements taken approximately every hour on the selected days.

### 2.4. Database

The database consisted of 1823 records, each representing a unique observation containing 11 variables collected throughout the study. These included four primary variables—pH, dissolved oxygen, temperature, and fish condition—along with several complementary variables such as the month, date, time, number of fish, number of plants, fish weight, and lettuce size. To ensure data integrity, quality control procedures were implemented to identify and correct outliers or inconsistencies, and the records were systematically categorized according to the type of variable monitored. Once validated and organized, the dataset was stored in a .csv file format, ensuring compatibility with statistical analysis software and predictive modeling tools. For subsequent analysis, the numerical variables (pH, dissolved oxygen, and temperature) were normalized using a min–max scaling method. After this preprocessing step, the resulting dataset had a final shape of 1823 rows and 10 columns.

## 3. Classification Model

The classification model aimed to recognize three distinct conditions in fish welfare: Condition 1 (Healthy—optimal state), Condition 2 (Stress—intermediate state), and Condition 3 (Mortality—critical state), based on features computed from variables such as dissolved oxygen, temperature, and pH. This task was framed as a multiclass classification problem, where each instance is assigned to one of the three predefined states. The variables pH, dissolved oxygen, and temperature were selected due to their critical influence on water quality and fish health, as documented in the aquaponics literature [14,26]. These parameters were measured using three distinct sensors integrated into the aquaponic system. Each input sample corresponds to an individual sensor reading taken at a specific time and is labeled according to the observed fish welfare condition. Before model training, the selected input variables were normalized using min–max scaling to transform their values into the [0, 1] range, ensuring consistent feature scales and improving model performance.

### 3.1. Classification Algorithms

To discriminate between fish welfare classes, linear discriminant analysis (LDA) was used as the classification algorithm [27]. This algorithm is straightforward to implement and yields good classification performance within the context of aquaponics systems [28]. It has also been successfully employed in prior studies [29]. The objective of LDA is to compute a separation hyperplane defined by $w^{T}\cdot x=0$, where $x\in R^{p\times 1}$ is the feature vector, and $w\in R^{p\times 1}$ is a weight vector to be calculated. Given a set of *N* training examples $\left\{\left(x_{i},y_{i}\right),i=1,\ldots ,N\right\}$, the weight vector is derived through the following optimization problem: [27]:

(1) $$w=\arg\max_{w}\frac{w^{T}S_{B}w}{w^{T}S_{W}w}$$

where $S_{B}$ is the between-class covariance matrix, and $S_{W}$ is the within-class covariance matrix [30]. Finally, the function that estimates the class for a new feature vector is given as follows:

(2) $$\hat{y}=sign\left(w^{T}x\right)$$

where $\hat{y}=\left\{1,2,3\right\}$ is the estimated class, corresponding to Condition 1 (Healthy—optimal state), Condition 2 (Stress—intermediate state), and Condition 3 (Mortality—critical state), respectively.

A support vector machine (SVM) was used for the classification task, as this algorithm has shown strong performance in applications involving aquaponic system data [31]. A support vector machine takes as its input a set of *n* feature vectors, $\vec{x_{i}}$, together with their labels, $y_{i}\in \left\{1,-1\right\}$. The idea behind SVMs is to find the hyperplane that maximizes the distance between the examples of the two classes, {1,−1}. This is done by finding a solution to the optimization problem

(3) $$\min_{\vec{w},b,\xi }\;C\sum_{i=1}^{n}\xi_{i}+\frac{1}{2}{∥\vec{w}∥}^{2}\;,$$

subject to the condition

(4) $$y_{i}\left({\vec{w}}^{T}\phi \left(\vec{x_{i}}\right)+b\right)\geq 1-\xi_{i}\;,$$

where $\vec{w}$ is the normal to the hyperplane, and $\xi_{i}\geq 0$ are slack variables that measure the error in the misclassification of $\vec{x_{i}}$.

Multilayer perceptron neural networks (MLPs) are composed of a computation unit called a perceptron, which is defined by Equation (5). Their objective is to separate two classes using a hyperplane.

(5) $$O\left(x\right)=F\left(\sum_{i=1}^{n}x_{i}w_{i}+b\right)$$

where $x_{i}$ represents the *i*th element of the input vector x. The synaptic weights are w, *b* is the bias, and *F* represents a non-linear activation function [32,33,34]. In our case, the softmax activation function is as follows:

(6) $$P\left(y=j|x\right)=\frac{e^{z_{j}}}{\sum_{k}e^{z_{k}}}$$

where $z_{j}$ is the output of the last hidden layer before softmax activation. A multilayer perceptron (MLP) network typically includes more than two hidden layers, each containing a variable number of perceptrons. These networks are trained using stochastic gradient descent.

Feedforward neural networks (FNNs) can be optimized using genetic algorithms (GAs) to improve both weight selection and architectural tuning of the network [35]. The GA starts by generating a population of individuals, where each individual represents a particular network configuration defined by the number of neurons in the hidden layers. In this case, the individual vectors are defined as

(7) $$g=[n_{1},n_{2}],$$

where $n_{1}\in \left[64,256\right]$ is the number of neurons in the first hidden layer, and $n_{2}\in \left[32,128\right]$ is the number of neurons in the second hidden layer.

Each individual is evaluated through a fitness function that calculates the classification accuracy achieved by the FNN model trained with that configuration. Formally, given a training set, $D_{train}={\left\{\left(x_{i},y_{i}\right)\right\}}_{i=1}^{M}$, and a test set, $D_{test}$, the model parametrized by g is trained to minimize the categorical cross-entropy loss function

(8) $$L=-\sum_{i=1}^{M}\sum_{c=1}^{C}y_{i,c}\log{\hat{y}}_{i,c},$$

where $y_{i,c}$ is the one-hot encoded true label, and ${\hat{y}}_{i,c}$ is the predicted probability for class *c*.

The fitness function f(g) is defined as the accuracy on the test set, evaluated after a preliminary training of 10 epochs:

(9) $$f\left(g\right)=\frac{1}{|D_{test}|}\sum_{\left(x_{j},y_{j}\right)\in D_{test}}I\left({\hat{y}}_{j}=y_{j}\right),$$

where I is the indicator function, and ${\hat{y}}_{j}$ is the predicted class for example *j*.

The algorithm evolves the population through genetic operators: tournament selection, blend crossover, and mutation, where mutation randomly modifies $n_{1}$ and $n_{2}$ within their permitted ranges to explore new configurations. Finally, the best configuration found, $g^{*}$, is trained for a larger number of epochs (500) to fine-tune the network weights and maximize performance.

This procedure ensures that the FNN architecture is optimally adapted to the classification problem of fish conditions based on dissolved oxygen, temperature, and pH measurements, thereby improving model accuracy and robustness.

Random forest is an ensemble learning method that combines multiple decision trees to improve classification accuracy and reduce overfitting [36,37]. Each tree, $T_{i}$, in the forest produces a prediction for the input feature vector x, and the final classification is obtained by aggregating these individual predictions. Mathematically, the predicted class $\hat{y}$ is given as follows:

(10) $$\hat{y}=\mathrm{mode}\left\{T_{1}\left(x\right),T_{2}\left(x\right),\ldots ,T_{N}\left(x\right)\right\}$$

where *N* is the total number of trees in the forest, and $T_{i}\left(x\right)$ denotes the prediction of the *i*-th decision tree for input x. For classification tasks that output probabilities, the aggregated probability for class *j* is as follows:

(11) $$P\left(y=j|x\right)=\frac{1}{N}\sum_{i=1}^{N}P_{i}\left(y=j|x\right)$$

where $P_{i}\left(y=j|x\right)$ is the predicted probability for class *j* via tree *i*.

In this study, fish conditions were predicted using a random forest model trained with physiological variables such as pH, dissolved oxygen, and temperature. The model was evaluated using metrics like accuracy and confusion matrices, demonstrating robust classification performance for the multi-class problem.

### 3.2. Model Training

Model training aimed to optimize the performance of multiple classification algorithms through iterative adjustment and validation. The models evaluated included linear discriminant analysis (LDA), support vector machines (SVMs), a neural network with a Softmax output layer, and random forest.

To prevent overfitting and ensure robust generalization, a 5-fold cross-validation strategy was applied uniformly across all models. This approach allowed each model to be trained and validated on multiple partitions of the training data, improving reliability in performance estimation.

Furthermore, to assess whether the models were capturing meaningful patterns, rather than learning from noise, a permutation test was implemented. This involved repeating the training and evaluation process using datasets with randomly shuffled labels, establishing a baseline to compare against the performance obtained under real conditions. A total of 10 permutations were conducted for each model, and each permutation was evaluated using 5-fold cross-validation, resulting in 50 evaluations per model with shuffled labels to ensure the stability of the baseline estimates.

The LDA and random forest models were trained directly on raw input data without normalization. The random forest used 100 estimators and default parameters for depth and feature selection. In contrast, the SVM and the neural network models used standardized inputs (z-score normalization). The SVM was configured with a radial basis function (RBF) kernel and trained using default parameters (C=1.0, γ=scale).

For the neural network, a sequential architecture was implemented with two fully connected hidden layers of 64 and 32 neurons, respectively, using ReLU activations, followed by a Softmax output layer for multiclass classification. The network was trained using the Adam optimizer, categorical cross-entropy loss, and a batch size of 32 for 50 epochs. Model weights were updated through backpropagation to minimize prediction error across iterations.

It is important to note that the cross-validation and permutation analyses described in this section were conducted as part of the training phase to ensure robustness and avoid overfitting. While Section 3.3 details the final 70/30 split applied to establish an independent test set, both approaches were complementary: cross-validation provided a reliable internal validation framework, whereas the hold-out split ensured a consistent and unbiased evaluation of model generalization.

### 3.3. Strategic Data Split

To ensure consistent evaluation and model comparability, the dataset was uniformly partitioned into 70% for training and 30% for testing across all machine learning algorithms. This split was chosen to provide sufficient data for learning while preserving an independent subset for assessing generalization performance. All models—linear discriminant analysis (LDA), support vector machines (SVMs), neural networks, and random forest—were trained using this same strategy, ensuring methodological consistency throughout the study.

### 3.4. Metrics for Evaluation

To comprehensively assess model performance, several evaluation metrics were employed. First, accuracy was used to measure the proportion of correctly classified instances, offering a general overview of the model’s effectiveness. However, since accuracy alone may be insufficient in imbalanced datasets, additional metrics were considered.

Specifically, precision and recall were used to evaluate the reliability of positive predictions and the model’s ability to identify all relevant instances, respectively. These two metrics were then combined into the F1-score, a harmonic mean that provides a balanced assessment, especially useful when trade-offs between precision and recall are relevant.

Furthermore, the receiver operating characteristic–area under the curve (ROC-AUC) was calculated to assess the model’s ability to discriminate between classes across different threshold settings. Together, these metrics provided a robust framework for comparing model performance and guiding the selection of the most suitable classification strategy.

## 4. Results

All analyses were conducted on Windows 10, 64-bit (platform string: Windows-10-10.0.19045-SP0), using Python 3.11.3 (Anaconda). The main libraries employed were NumPy 1.24.3, Pandas 1.5.3, Matplotlib 3.7.1, Seaborn 0.12.2, SciPy 1.10.1, Scikit-learn 1.2.2, and TensorFlow 2.17.0 (Keras API). The hardware configuration consisted of an Intel(R) Core(TM) i5-9300H CPU @ 2.40 GHz, 8 GB RAM, a 238 GB SSD NVMe (ADATA SX6000PNP), and the graphics adapters NVIDIA GeForce GTX 1650 (4 GB) and Intel(R) UHD Graphics 630.

### 4.1. Analysis of Variables from Fish Conditions

The distributions of three key environmental variables—pH, dissolved oxygen, and temperature—were analyzed as a function of fish condition, which was categorized into three classes: Condition 1 (Healthy—optimal state), Condition 2 (Stress—intermediate state), and Condition 3 (Mortality—critical state). These classifications were established based on systematic observations conducted throughout the production cycle.

The stress condition was assigned when fish exhibited repeated surface gasping episodes lasting at least 15 min in two or more consecutive observations, spaced by 30 min. Additional behavioral signs included erratic swimming, a loss of schooling behavior, and a diminished feeding response, consistent with validated welfare indicators for tilapia under suboptimal water quality conditions [4,7,38].

Mortality was confirmed by complete cessation of movement and opercular activity, verified visually by two independent observers. Although post mortem necropsies were not conducted, the absence of external lesions or visible disease suggested environmental causes, particularly hypoxia or temperature-related stress. The Healthy condition was characterized by normal swimming, group cohesion, and active feeding, with no visible signs of distress.

#### 4.1.1. Distribution of pH

**Figure 2:**
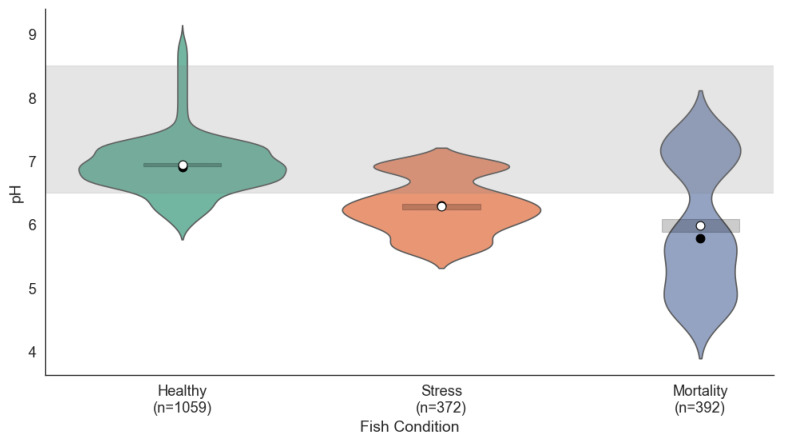
pH distribution across the three fish conditions: Condition 1 (Healthy), Condition 2 (Stress), and Condition 3 (Mortality).

Figure 2 shows the pH distribution across the three fish conditions. The pH distribution revealed notable differences among the three fish conditions. In the Healthy condition, pH values were concentrated between 7.0 and 7.5, falling within the ideal range (6.5–8.5), which indicates a stable and favorable chemical environment for the fish. Under Stress conditions, the pH values exhibited greater dispersion, ranging from 6.5 to 7.8; although mostly within the acceptable range, such fluctuations could contribute to stress. In the Mortality condition, even greater dispersion was observed, with values approaching the lower limit of the ideal range, suggesting that inadequate pH control may have significantly contributed to fish mortality.

To statistically validate these differences, a one-way ANOVA test was performed, revealing a significant effect of fish condition on pH values (F=405.68, p<0.001). A subsequent Tukey HSD post hoc analysis confirmed that all pairwise comparisons were significantly different (adjusted p<0.001), with mean differences of –0.15 (Healthy–Stress), –0.22 (Healthy–Mortality), and –0.07 (Stress–Mortality). These findings reinforce the relevance of pH as a sensitive indicator of fish health.

#### 4.1.2. Dissolved Oxygen Distribution

**Figure 3:**
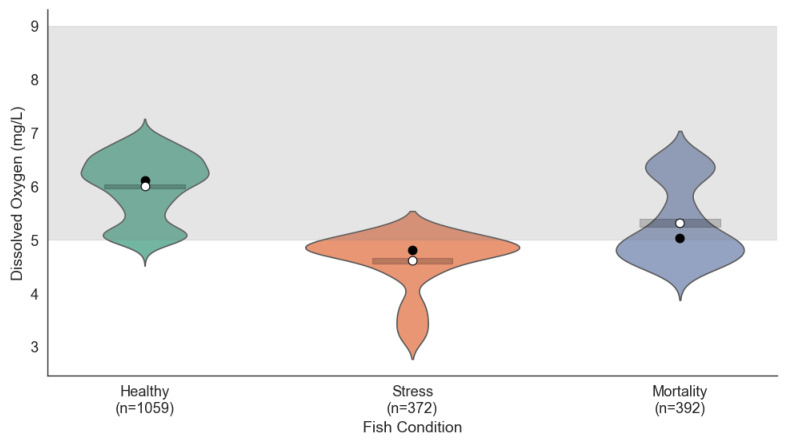
Dissolved oxygen distribution for Healthy, Stress, and Mortality fish conditions.

Dissolved oxygen levels also showed clear differences across the three fish conditions (see Figure 3). In the Healthy condition, values were maintained between 6 and 7 mg/L, within the optimal range (5–8 mg/L), ensuring adequate oxygenation for the fish. Under Stress conditions, oxygen levels dropped to between 4 and 5 mg/L, which may be insufficient to meet the physiological needs of the fish, leading to stress. In the Mortality condition, critical levels close to 4 mg/L were observed, indicating severe hypoxia as a likely primary cause of fish mortality.

ANOVA results for dissolved oxygen confirmed statistically significant differences among the three conditions (F=765.38, p<0.001). Tukey’s post hoc test revealed that all group comparisons were significantly different (adjusted p<0.001), with mean differences of −0.36 (Healthy–Stress), –0.18 (Healthy–Mortality), and 0.18 (Stress–Mortality). These results underscore dissolved oxygen as a critical environmental factor associated with fish stress and mortality.

#### 4.1.3. Temperature Distribution

**Figure 4:**
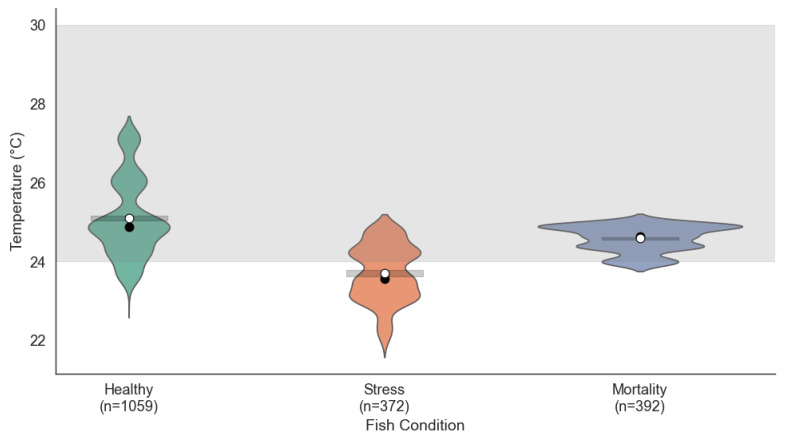
Temperature distribution across the three fish conditions: Condition 1 (Healthy), Condition 2 (Stress), and Condition 3 (Mortality).

Temperature patterns also varied according to the fish condition (see Figure 4). In the Healthy condition, values were concentrated between 24.5 °C and 26.5 °C, within the ideal range of 24 °C to 30 °C, indicating effective thermal regulation. Under stress conditions, the temperature showed less variation, ranging from 23.5 °C to 25 °C. Although close to the ideal range, these lower temperatures could create suboptimal conditions for the fish. In the mortality condition, the temperature distribution remained stable but was concentrated at the lower limit of the ideal range (24 °C to 25 °C), suggesting that sustained exposure to these temperatures may have contributed to the decline in fish health.

The ANOVA test for temperature revealed significant differences across the fish condition groups (F=392.03, p<0.001). According to the Tukey HSD test, all pairwise comparisons were statistically significant (adjusted p<0.001), with mean differences of –0.27 (Healthy–Stress), –0.10 (Healthy–Mortality), and 0.17 (Stress–Mortality). In terms of variability, the temperature variance was highest in Healthy fish (0.036), intermediate in stress (0.019), and lowest in mortality (0.004), supporting the statement that temperature fluctuations were more constrained under mortality events. These results reinforce the role of temperature as a modulating factor in fish health within aquaponic environments.

### 4.2. Classification

**Figure 5:**
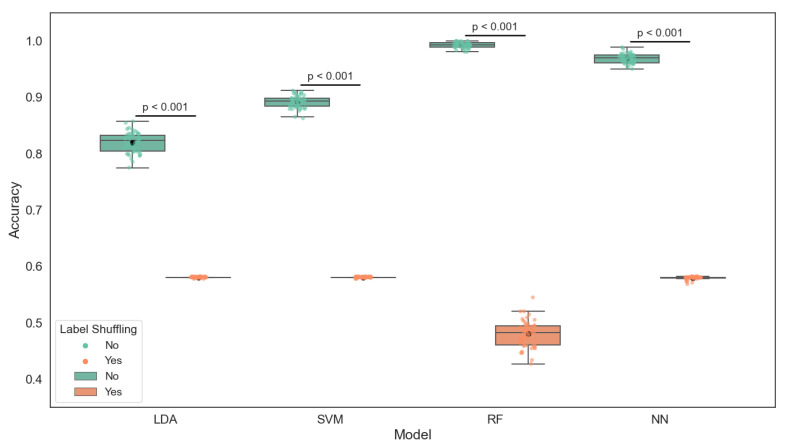
Comparison of classification accuracy across models under real and randomized label conditions. Accuracy values were obtained from 10 repetitions of 5-fold stratified cross-validation for each model. Individual points represent accuracy per fold and repetition. Box plots show the distribution of accuracies, with the mean indicated by a black circle. Horizontal lines above each pair of boxes indicate the p-values from paired *t*-tests comparing performance with real versus shuffled labels.

As illustrated in Figure 5, the boxplot presents the classification accuracy of four machine learning models—linear discriminant analysis (LDA), support vector machine (SVM), neural network (NN), and random forest (RF)—under two conditions: using true labels and using randomly shuffled labels. These models were trained on key water quality indicators, including pH, dissolved oxygen, and temperature, to predict fish health status categorized into Healthy, Stress, and Mortality.

The results clearly show that all models perform significantly better with true labels, as supported by independent *t*-tests (all p<0.0001). Among the models, random forest achieved the highest mean accuracy (0.99), followed by the neural network (0.97) and SVM (0.89). LDA yielded the lowest, though still acceptable, accuracy (0.82). The substantial drop in accuracy when labels are shuffled highlights that the models are not overfitting noise but are indeed learning meaningful relationships from the input data. These findings confirm the predictive power of the selected water quality parameters in assessing fish condition within the aquaponic system.

The classification performance of the evaluated models is summarized in Table 2, which reports the mean accuracy under both real and shuffled label conditions, the statistical significance (*p*-value) of the performance difference, and the detailed precision, recall, and F1-score per class.

A total of 1823 labeled instances were collected during the monitoring period, distributed as follows: 1059 labeled as Healthy, 372 as Stress, and 392 as Mortality. Stratified 5-fold cross-validation was used for model training and evaluation. Table 2 shows performance metrics on a representative test subset (n=547), drawn from one of the folds and preserving class proportions (310 Healthy, 115 Stress, 122 Mortality). This class distribution is moderately imbalanced, especially between the healthy and stress categories, which is relevant when interpreting metrics such as recall and F1-score.

To evaluate whether the observed model performance was significantly better than random chance, we conducted a permutation test comparing two conditions: models trained with the true labels and models trained with randomly shuffled labels (10 repetitions). In both cases, training and evaluation followed the same 5-fold cross-validation procedure. The resulting accuracy distributions were compared using a two-sample independent *t*-test, with all models showing statistically significant differences (*p* < 0.001), indicating that performance was not attributable to label noise or random alignment.

Given the imbalance between classes—particularly the underrepresentation of Stress (n=372) and Mortality (n=392) compared to Healthy (n=1059)—we included per-class precision, recall, and F1-score to evaluate how well each model detects critical conditions. For example, LDA achieved 0.95 recall for Healthy but only 0.47 for Mortality, while random forest maintained a recall above 0.97 for all classes. These disparities underscore the need to report more than global accuracy when evaluating predictive models in unbalanced real-world settings.

Models must be robust not only in detecting the majority class but also in correctly identifying minority and critical conditions, especially in applications where the early detection of stress or mortality is crucial for system intervention. The input features consisted of pH, dissolved oxygen, and temperature—parameters critical for assessing water quality in aquaponic environments.

The linear discriminant analysis (LDA) model obtained a mean accuracy of 0.820 under real labels and 0.581 with shuffled labels, yielding a statistically significant (*p*< 0.001). LDA showed strong performance in detecting healthy fish (Healthy Class), achieving a recall of 0.95 and F1-score of 0.87. However, performance decreased substantially for Mortality Class (Mortality), with a recall of only 0.47, highlighting limitations in identifying critically affected fish.

The support vector machine (SVM) model achieved a mean accuracy of 0.892 under real conditions and 0.581 when labels were shuffled (*p* < 0.001), indicating significant predictive power. The Healthy class reached a recall of 0.99 and F1-score of 0.91, while the Stress class exhibited robust classification with an F1-score of 0.95. However, the Mortality class continued to show weaker results, with a recall of 0.55.

The neural network (softmax) achieved a mean accuracy of 0.967 under real conditions, while performance dropped to 0.580 when trained on shuffled labels (*p* < 0.001). This model maintained consistently high precision, recall, and F1-scores across all classes. The Healthy class and Stress class metrics were close to 0.99 and 0.97, respectively, while the Mortality class also demonstrated solid performance with an F1-score of 0.95.

Among all evaluated models, the random forest classifier yielded the highest accuracy, with 0.993 for real labels and 0.481 under shuffled conditions (p<0.001). It demonstrated excellent generalization across all classes, particularly for the Healthy class and the Stress class, both nearing perfect scores in all metrics. Notably, the Mortality class also achieved a high F1-score of 0.98, confirming the model’s robustness in detecting critical health conditions.

**Figure 6:**
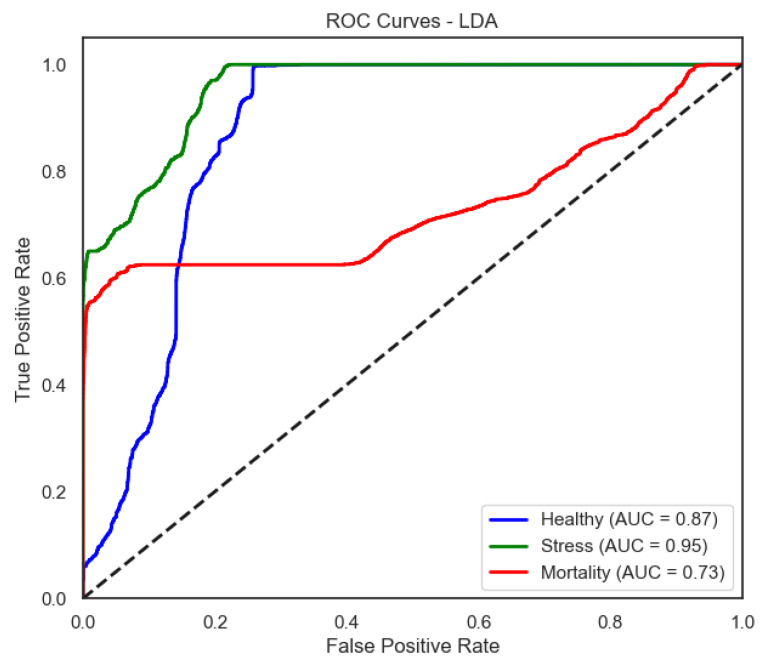
ROC curve of the LDA model (one-vs-rest).

**Figure 7:**
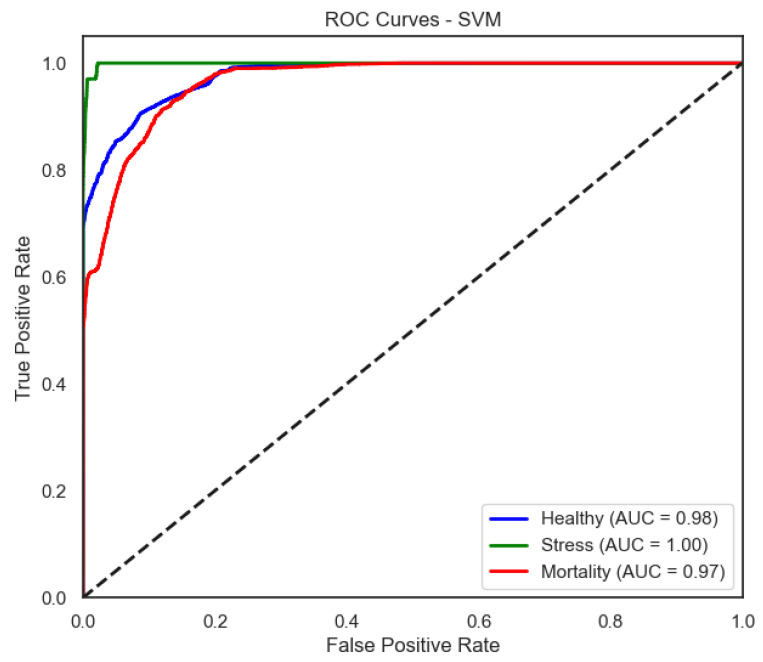
ROC curve of the SVM model (one-vs-rest).

**Figure 8:**
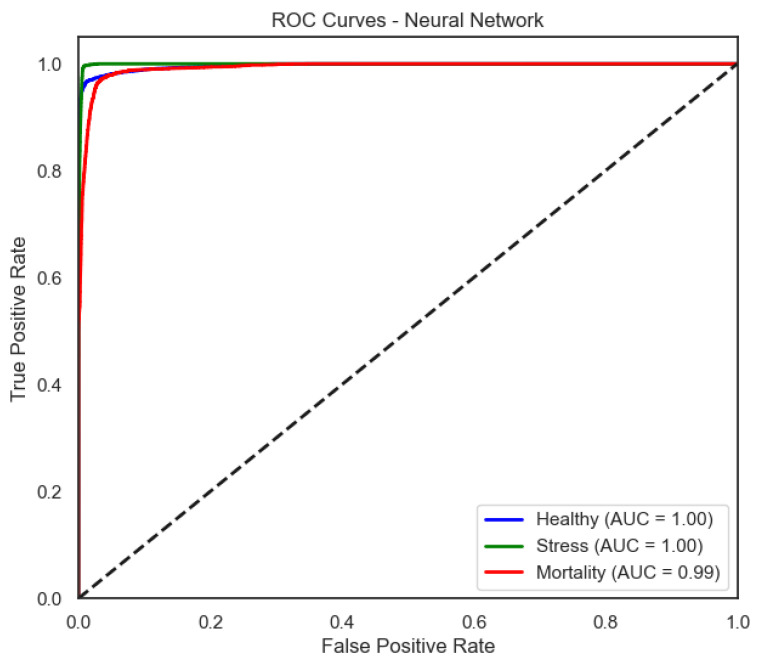
ROC curve of the Neural Network model (one-vs-rest).

**Figure 9:**
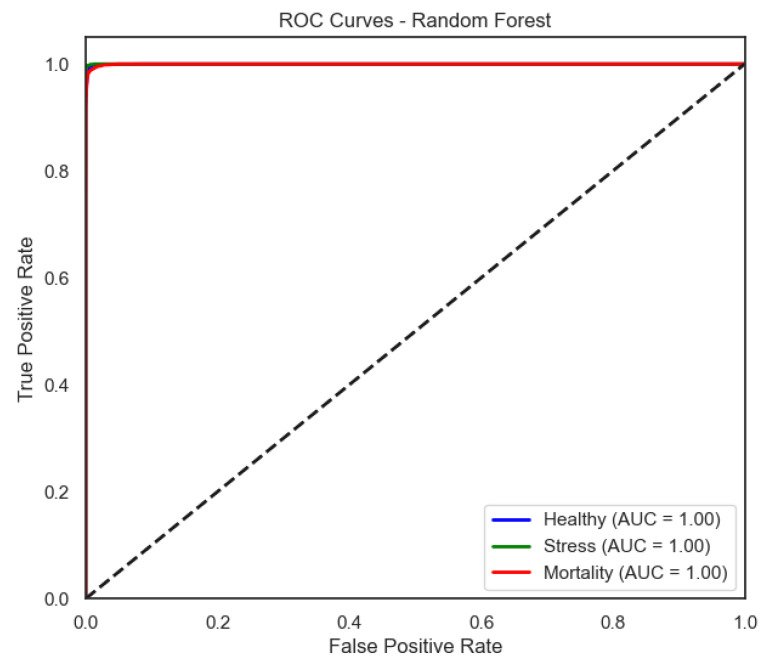
ROC curve of the Random Forest model (one-vs-rest).

The ROC curves (Figure 6, Figure 7, Figure 8 and Figure 9) provide a complementary view of classification performance. Consistent with the accuracy metrics, random forest and the neural network exhibited areas under the curve (AUC) close to 1.0 across all classes, confirming their robustness. In contrast, LDA showed reduced separability for the Mortality class, reinforcing the limitations highlighted in its recall and F1-score.

For LDA, the ROC curves show moderate separability between classes, with weaker discrimination for Mortality—aligned with its lower recall for that class.

SVM improves the separation, particularly for Stress and Healthy, mirroring its higher F1-score in these categories.

The neural network exhibits consistently high true positive rates across thresholds, indicating stable discrimination for all classes.

Random forest approaches near-perfect separability, in line with its near-perfect per-class precision/recall.

We also provide the normalized confusion matrices to visualize error patterns by class. These plots clarify where misclassifications occur and complement precision/recall values.

**Figure 10:**
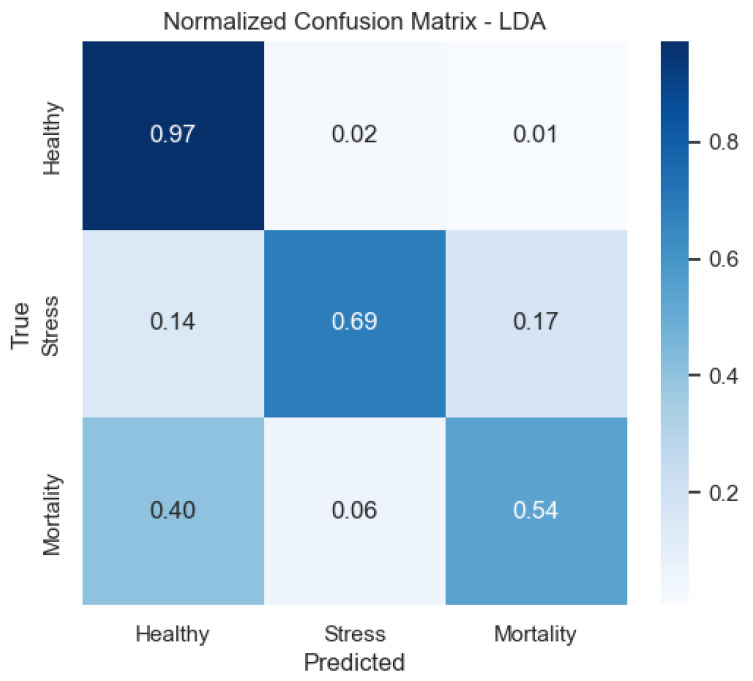
Confusion matrix of the LDA model (row-normalized).

As shown in Figure 10, in the LDA model Mortality is the most frequently confused class, whereas Healthy is well identified—consistent with its recall profile.

**Figure 11:**
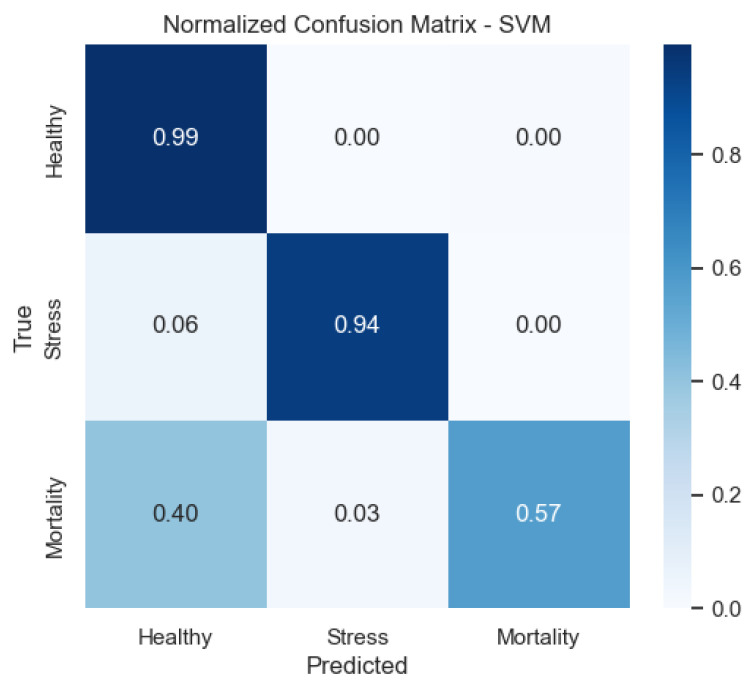
Confusion matrix of the SVM model (row-normalized).

Figure 11 shows that the SVM reduces confusion between Stress and Healthy compared with LDA, but still shows some spillover from Mortality.

**Figure 12:**
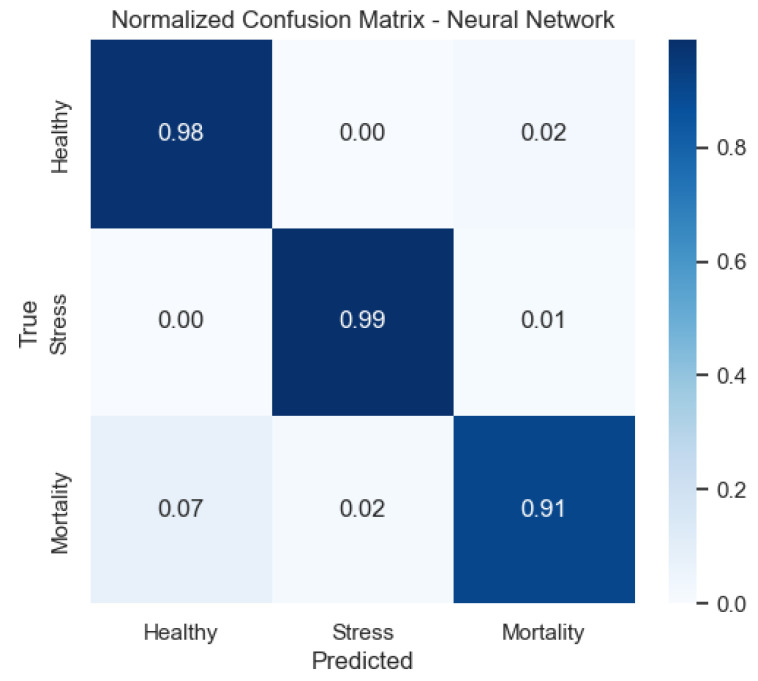
Confusion matrix of the Neural Network model (row-normalized).

Figure 12 demonstrates that the neural network achieves balanced detection across categories, with limited off-diagonal mass.

**Figure 13:**
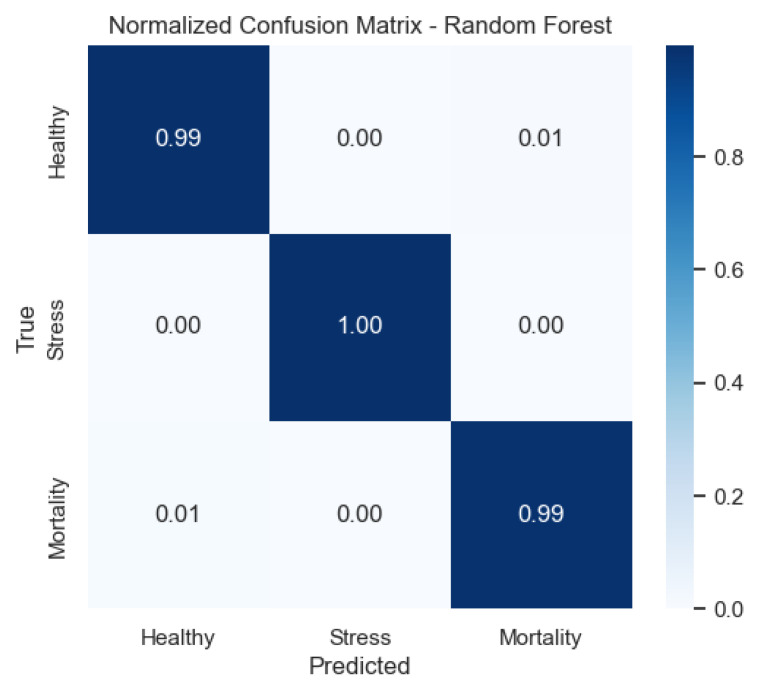
Confusion matrix of the Random Forest model (row-normalized).

Figure 13 illustrates that the random forest shows near-diagonal matrices, indicating minimal confusion and corroborating its superior F1-scores.

**Figure 14:**
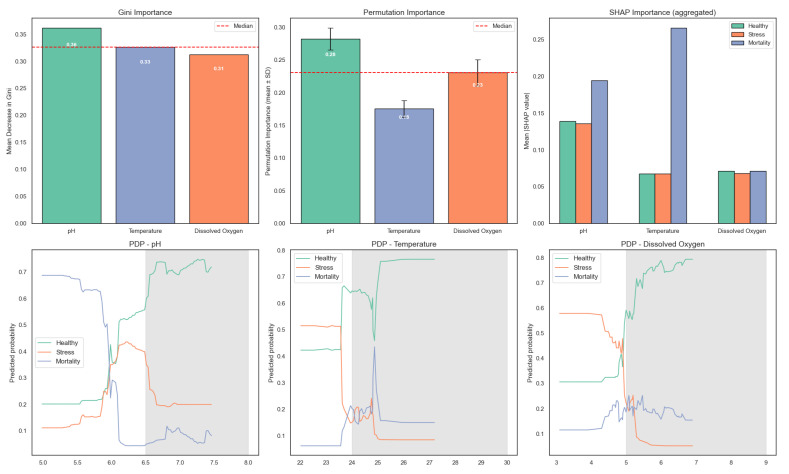
Feature importance of the random forest model for predicting fish physiological states in aquaponic systems. The first subplot shows Gini importance, highlighting pH as the most influential variable, followed by dissolved oxygen and temperature. The second subplot shows permutation importance (mean ± SD), confirming a similar ranking. The third subplot displays aggregated SHAP values across all classes. The bottom row shows partial dependence plots (PDPs) for the top three features, with recommended ranges shaded.

To further interpret the behavior of the random forest model, the relative contribution of each environmental variable was analyzed using the mean decrease in Gini impurity. The contribution of each input variable to the random forest model is illustrated in Figure 14. pH exhibited the highest importance, followed by dissolved oxygen and temperature. These results are consistent across Gini importance, permutation importance (with confidence intervals), and SHAP values, further supporting the model’s capacity to distinguish fish physiological states based on key environmental variables. Partial dependence plots reveal the marginal effects of each variable on predicted probabilities for Healthy, Stress, and Mortality classes.

This variable ranking aligns with the statistical findings from the ANOVA and Tukey’s HSD tests, which showed significant differences in dissolved oxygen and temperature across fish condition categories. The agreement between statistical and model-based analyses reinforces the importance of these parameters in predicting stress and mortality in aquaponic systems.

## 5. Conclusions and Discussion

This study demonstrated the effectiveness of machine learning algorithms in classifying the physiological condition of red tilapia (*Oreochromis* spp.) within an aquaponic system, using key environmental variables such as temperature, pH, and dissolved oxygen. The models accurately distinguished between three health states—Healthy, Stress, and Mortality—based on real-world data collected from the aquaponic environment.

Among the evaluated classifiers, the random forest algorithm achieved the highest overall performance, with an accuracy of 0.99. This result is explained by its ensemble structure, which captures non-linear interactions and complex patterns in environmental data more effectively than LDA or SVM. Its robust generalization and reduced overfitting make it particularly suitable for environments with fluctuating water quality, as commonly seen in aquaponic systems.

A statistical analysis using ANOVA and Tukey’s HSD test confirmed that pH, temperature, and dissolved oxygen vary significantly across fish condition categories. Notably, dissolved oxygen and temperature showed the largest mean differences, reinforcing their critical role as indicators of fish welfare in aquaponics.

The model goes beyond classification by integrating probability-based alerting. This allows it to effectively distinguish between high-risk and low-risk scenarios for stress and mortality. The probabilistic framework is well suited for real-time monitoring, enabling timely interventions and improving fish welfare.

Crucially, this model is both theoretically solid and practically implementable. It can be deployed on low-cost embedded systems (e.g., Raspberry Pi, ESP32) together with digital sensors, enabling automated regulation of aeration, heating, or nutrient dosing once risk thresholds are detected. This represents a substantial advancement over manual or simplistic automated management typically used in aquaponic setups [13,18].

Compared to previous studies that focused on sensor integration or basic control systems [16,18], our work delivers a fully integrated solution: a predictive model trained on field data, an alerting mechanism, and deployment-ready design for embedded systems. By explicitly targeting early detection of stress and mortality in fish—a focus largely absent from earlier literature—this research addresses a critical gap and contributes a practical path toward sustainable and intelligent aquaponic management.

By enabling early stress detection and preventing mortality, the system directly targets key challenges in aquaponics—preserving animal welfare while ensuring water quality. This enhances resource efficiency and aligns with global goals of food security and climate resilience.

Despite the promising results obtained, the dataset presented a natural class imbalance, with Healthy cases (n=1059) outnumbering Stress (n=372) and Mortality (n=392). To mitigate potential bias in performance assessment, the evaluation relied on class-specific metrics (precision, recall, and F1-score), allowing for a balanced interpretation across categories. Alternative resampling strategies, such as oversampling or undersampling, were deliberately avoided to preserve the original distribution and prevent artificial alterations to the biological patterns observed in the system. Future studies could explore the use of such resampling techniques to investigate their potential impact on predictive performance.

All sensors were calibrated weekly according to manufacturer guidelines. The PH4502C pH sensor was adjusted using a potentiometer (2.5V in pH 7 buffer) and calibrated with pH 4.0 and 7.0 standards. The DS18B20 temperature sensor was verified against a glass thermometer. The 183–ODPORT dissolved oxygen sensor, factory-calibrated and certified, was occasionally cross-checked with a YSI meter. Manufacturer-reported accuracies are ±0.1–0.2 pH units for pH, ±0.5 °C for temperature, and ±0.3mg/L for dissolved oxygen, supporting reliable and traceable measurements.

Although the sensors provided reliable accuracy during this study, a formal uncertainty propagation into the classifier outputs was not conducted. Future work will explicitly address this aspect to quantify how sensor measurement errors influence predictive reliability, thereby strengthening the robustness of the proposed monitoring framework.

It is important to note that stress and mortality events in this study emerged spontaneously under real aquaponic operating conditions. No artificial perturbations (e.g., deliberate reduction in dissolved oxygen) were introduced to provoke system failures or extreme responses. This decision was guided by ethical considerations and a desire to maintain ecological validity. Nonetheless, the absence of controlled disturbances may limit the model’s ability to generalize to rare but critical events. Future research should consider experimentally inducing stressors under controlled conditions to assess model robustness in high-risk scenarios and expand its applicability to a wider range of aquaponic environments.

While the model demonstrated strong performance in predicting fish health conditions within the red tilapia–lettuce aquaponic system, its applicability to other species or crop types has not yet been tested. Physiological tolerances—particularly to dissolved oxygen and temperature—can vary substantially across fish species. For example, species such as trout or catfish have different metabolic rates and oxygen demands compared to tilapia. These interspecies differences may limit direct model migration and warrant retraining or recalibration when applied to alternative configurations. Moreover, environmental factors such as temperature fluctuations or water recirculation dynamics may affect model reliability under different climate conditions. Although the current system was tested in a controlled aquaponic environment, future work will focus on evaluating the model’s generalizability by incorporating data from systems involving diverse fish species, plant configurations, and geographical contexts. This strategy aligns with the goal of developing scalable and adaptive AI-driven monitoring tools, as discussed by previous studies on embedded system deployment and multi-environment validation [13,14,18].

It is important to acknowledge that fish classification in this study was conducted through visual observation, without necropsy or biochemical markers. However, visual inspection remains a widely used, practical approach for on-site welfare assessment in aquaculture, especially when conducted by trained personnel [4,5,7,38]. Observable indicators such as abnormal swimming, changes in coloration, and surface gasping have been validated as early signs of stress or disease. Future research will seek to incorporate clinical validation (e.g., blood chemistry or histopathology) to further refine the training labels and ensure compliance with evolving welfare standards.

While formal inter-rater agreement (Cohen’s κ) was not calculated for this dataset, both observers reconciled any discrepancies through discussion, ensuring consistent labeling. Future campaigns may include formal κ assessment or non-invasive physiological proxies to further validate label robustness under ethical constraints.

Future work will explore the integration of SHAP-based interpretability methods and external validation on independent datasets to further strengthen the model’s transparency and generalizability.

In comparison with previous studies on AI applications in aquaculture, our approach offers distinct and practical contributions. While many prior efforts emphasize image-based monitoring systems, such as machine vision or video-based welfare assessment [19,20], these often require complex infrastructure and high computational resources, limiting field deployment. In contrast, our system relies on real-time sensor data (pH, temperature, dissolved oxygen) and embedded machine learning models, providing an affordable and easily deployable solution for small-scale producers. Other recent works have focused on water quality prediction using deep learning or IoT frameworks [14,39], yet few have tackled the early detection of physiological distress or mortality with interpretable outputs tailored to aquaponic settings. By incorporating explainable models like Random Forest and LDA, our system not only predicts adverse conditions but also supports decision-making by highlighting critical variables. Thus, this work fills a relevant gap by advancing both practical implementation and scientific understanding of AI-based monitoring in aquaponics.

The selection of pH, temperature, and dissolved oxygen as primary monitoring variables is not only rooted in practicality but also in robust physiological evidence. These parameters have a direct influence on metabolic efficiency, stress modulation, and survival outcomes in aquatic species, including tilapia, particularly under recirculating aquaponic systems [40,41]. Furthermore, the interaction between pH and temperature is critical for determining the proportion of un-ionized ammonia (${\mathrm{NH}}_{3}$), the toxic form of total ammonia nitrogen, which can be lethal even at low concentrations [42]. Therefore, by continuously monitoring pH and temperature, the system indirectly anticipates ammonia toxicity risks—especially relevant in closed-loop systems with limited water exchange. This approach has been widely validated in empirical studies and aquaculture practice, where these three parameters are considered foundational for maintaining fish welfare and the early detection of physiological distress [4,5,7].

Our methodology also aligns with technological trends in smart aquaponic systems, which prioritize the integration of low-cost, high-frequency sensors for these variables due to their high signal value in real-time health inference [13].

Although this study confirmed high predictive accuracy under controlled aquaponic operation, sustained deployment in real-world contexts demands an additional layer of evaluation. Long-term system reliability can be influenced by biofouling, sensor drift, and gradual mechanical wear, which may affect signal stability and, consequently, model performance. These risks are amplified in continuous recirculating systems, where sensor exposure is constant. To address these challenges, future work will focus on the following: (1) multi-season deployment to evaluate predictive consistency across environmental fluctuations, (2) establishing preventive maintenance protocols for regular sensor cleaning and calibration, and (3) monitoring system performance decay to develop adaptive retraining strategies. Implementing these measures in extended field trials will safeguard predictive accuracy over multiple production cycles and strengthen operational resilience, a critical factor for scaling AI-based aquaponic monitoring beyond experimental settings.

Although the current model was validated under controlled aquaponic operation, future research will extend its scope through multi-source data integration and multi-season trials to verify stability under varying environmental conditions. Additional water quality metrics, particularly direct measurements of total ammonia nitrogen, will be considered to complement the current feature set. These steps, combined with preventive maintenance and recalibration protocols, aim to ensure consistent performance and facilitate scaling to commercial aquaponic operations.

Environmental variability was not experimentally induced in this study. No deliberate manipulations—such as forced oxygen depletion, abrupt pH alteration, or chemical dosing—were applied to provoke stress or mortality events. All observed stress or mortality cases arose spontaneously under normal aquaponic operation. Routine husbandry actions, such as filter cleaning, were only performed *after* early signs of stress were detected, in compliance with standard welfare and ethical guidelines for aquaculture [43]. Consequently, the training dataset includes both pre- and post-intervention measurements, reflecting real-world operational conditions. While these actions may influence subsequent environmental measurements, they preserve ecological validity by capturing cause–effect dynamics naturally encountered in sustainable aquaponic management.

Future work should focus on implementing a closed-loop control architecture that integrates the predictive model with real-time feedback mechanisms. In addition to detection, the system could evolve toward prescriptive functionality, automatically recommending specific corrective actions, for example, increasing aeration rates, adjusting feeding frequency, or stabilizing pH through dosing agents such as bicarbonate or organic acids, based on the predicted condition and dominant environmental triggers. Validation across different species, plant configurations, and climatic conditions will strengthen generalizability. Long-term deployment will also assess adaptability to seasonal and operational variability.

Future developments should prioritize the definition of alert thresholds that maximize sensitivity to early signs of stress, while also quantifying the advance warning time that such thresholds can provide before severe conditions occur. Another promising direction is the incorporation of recent temporal patterns, extracted from short sliding windows of sensor data, to improve the anticipation of state transitions in aquaponic systems.

In conclusion, applying machine learning, particularly random forest, offers a transformative approach to intelligent aquaponic management. Its ability to predict stress and mortality accurately supports timely decision-making and lays the foundation for autonomous, responsive, and sustainable aquaponic systems.

## Figures and Tables

**Table 1:** Specifications of sensors and system parameters in the aquaponic setup.

Component	Model	Spec.	Notes
**Sensors**			
pH probe	PH4502C	0.01 pH; ±0.1–0.2	Weekly calib. (pH 4/7), ∼30 s, 2.5 V adj., cross-check
Temp. sensor	DS18B20	0.0625 °C; ±0.5 °C	Verified vs. glass thermometer, <1 s stable
DO probe	183–ODPORT	0.1 mg/L; ±0.3 mg/L	Factory calib., YSI check, ∼60 s, electrode cleaning
**System parameters**			
Tank volume	–	800 L	Single tank
Flow rate	–	∼180 L/h	Turnover ∼4.4 h
Water exch.	–	–	Only evap. replenishment
Sampling freq.	–	1/h	1823 points in 8 months

**Table 2:** Accuracy comparison and performance metrics of classification models for fish condition prediction (test subset, *n* = 547). Mean accuracy is reported with standard deviation.

	Overall Accuracy			Healthy			Stress			Mortality		
Model	Real	Shuffled	* p * -Value	Precision	Recall	F1-Score	Precision	Recall	F1-Score	Precision	Recall	F1-Score
LDA	0.820 ± 0.018	0.581 ± 0.001	<0.001	0.81	0.95	0.87	0.77	0.71	0.74	0.74	0.47	0.57
SVM	0.892 ± 0.011	0.581 ± 0.001	<0.001	0.83	0.99	0.91	0.97	0.92	0.95	0.97	0.55	0.70
NN	0.965 ± 0.011	0.581 ± 0.001	<0.001	0.99	0.99	0.99	0.97	0.97	0.97	0.94	0.95	0.95
Random Forest	0.993 ± 0.005	0.480 ± 0.019	<0.001	0.99	1.00	0.99	1.00	0.99	0.99	0.99	0.97	0.98

## Data Availability

The data generated and analyzed during this study are not publicly available in order to reserve their use for future publications. However, they are available from the corresponding author upon reasonable request.

## Abbreviations

The following abbreviations are used in this manuscript:

AI	Artificial Intelligence
APC	Article Processing Charge
DO	Dissolved Oxygen
LDA	Linear Discriminant Analysis
ML	Machine Learning
RF	Random Forest
SVM	Support Vector Machine
NN	Neural Network
pH	Potential of Hydrogen
RMSE	Root Mean Square Error
